# 1411. Missed HIV Clinic Visits among Youth Living with HIV in Alabama

**DOI:** 10.1093/ofid/ofac492.1240

**Published:** 2022-12-15

**Authors:** Jiaying Hao

**Affiliations:** University of Alabama at Birmingham, Vestavia Hills, Alabama

## Abstract

**Background:**

Adolescents and young adults with HIV consistently account for about one-fifth of new infections in the United States. YLWH have lower rates of testing, diagnosis, treatment engagement, and viral suppression than adults with HIV. YLWH are particularly challenged with missing medical appointments. Few studies have been published on youth living with HIV (YLWH)’s missed clinic visits and risk factors. Therefore, this study was designed to explore:

1). if the missed visits have significant associations with HIV treatment outcomes (more than 50 counts of CD4 counts drop, viral load increment, and viral suppression) in YLWH; 2). What factors are associated with missed visits for YLWH? 3) Are these factors different from the risk factors of all PLWH?

**Methods:**

A retrospective study using data records from UAB Family Clinic was designed. The study used the records from March 1^st^ 2020 to August 31st, 2021. Patients aged 16-24 were considered as YLWH. The primary outcomes are missed visits and treatment outcomes. CD4 change, Viral suppression and VL change during the study period were investigated for identifying the association between missed visit and treatment outcome. Age, gender, race, HIV transmission mode, insurance type and the viral suppression in index record were used as factors to predict missed visits. Chi-square test and logistic regression have been conducted in SAS 9.4.

**Results:**

YLWH with missed visits had more viral load rebound compared to those without missed visits (p=0.04).

For YLWH, gender and viral suppression in the index record have significant associations with missed visits (Gender: p=0.03, Viral suppression in index record: p=0.04).

In tests among all PLWH, Race (p=0.03), Insurance type (p=0.01) and viral suppression in index record (p=0.001) are associated with missed visits.

**Table 1.**
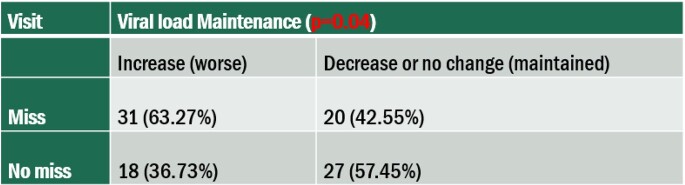
Viral load change by missed visits.

**Table 2.**
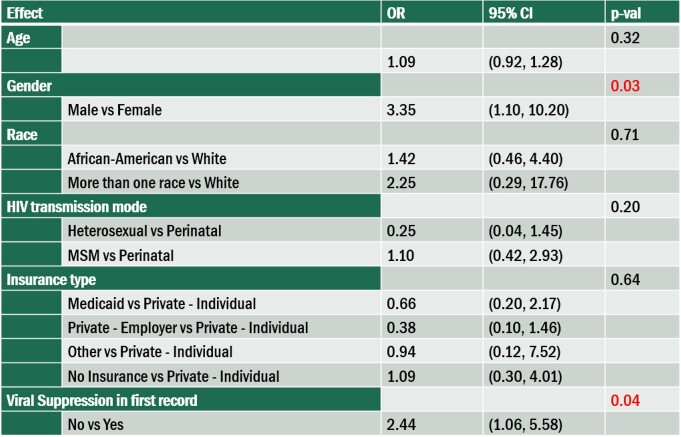
Risk factors associated with missed visits among YLWH (n=101).

**Table 3.**
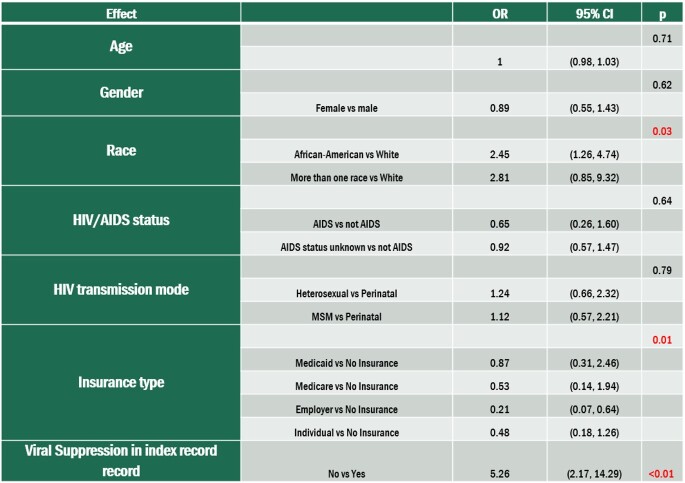
Risk factors associated with missed visits among PLWY (n=296).

**Conclusion:**

Missed visits are significantly associated with viral load maintenance, and missed visits have adverse effects on treatment outcomes. Also, the risk factors for YLWH of missed visits are not identical with the risk factors for PLWH from the same cohort.

**Disclosures:**

**All Authors**: No reported disclosures.

